# Contextualizing the Impostor “Syndrome”

**DOI:** 10.3389/fpsyg.2020.575024

**Published:** 2020-11-13

**Authors:** Sanne Feenstra, Christopher T. Begeny, Michelle K. Ryan, Floor A. Rink, Janka I. Stoker, Jennifer Jordan

**Affiliations:** ^1^Department of Human Resource Management and Organisational Behaviour, University of Groningen, Groningen, Netherlands; ^2^Psychology, University of Exeter, Exeter, United Kingdom; ^3^International Institute for Management Development, Business School, Lausanne, Switzerland

**Keywords:** impostor syndrome, impostor phenomenon, social context, stereotypes, institutional underrepresentation, unequal treatment

## Abstract

The impostor “syndrome” refers to the notion that some individuals feel as if they ended up in esteemed roles and positions not because of their competencies, but because of some oversight or stroke of luck. Such individuals therefore feel like frauds or “impostors.” Despite the fact that impostor feelings are often linked to marginalized groups in society, to date, research predominantly approaches this phenomenon as an issue of the individual: pointing toward individuals for the roots and solutions of the “syndrome.” Drawing from a rich body of social and organizational psychology research, in this perspectives piece, we propose a shift in how scholars conceptualize and empirically examine this phenomenon. Instead of framing the insecurities of individuals belonging to marginalized groups solely as a problem that arises *within* these individuals, we argue that it is critical for future research to consider the important role of the environment in eliciting their impostor feelings as well. By doing so, we can address the contextual roots of individuals’ impostor feelings, and offer more structural and effective solutions.

## Introduction

Many successful people, from former first lady Michelle Obama, to Facebook COO Sheryl Sandberg, have expressed feeling like impostors. Such admissions typically describe feelings of having ended up in esteemed roles not because of merits or achievement but because of some oversight on the part of important gatekeepers, or due to sheer luck. Despite their objective success, these individuals express difficulty internalizing their achievements and accomplishments and worry that they may be uncovered as frauds. This feeling is often referred to as the “impostor phenomenon*”* or “impostor syndrome” (Clance and Imes, 1978; Harvey, 1981; Bravata et al., 2019), and abundant research has shown its detrimental consequences for individuals’ well-being (e.g., Sonnak and Towell, 2001; McGregor et al., 2008) as well as career advancement (e.g., Kets de Vries, 2005; Vergauwe et al., 2014; Neureiter and Traut-Mattausch, 2016).

Over the last decade, attention toward this topic, and the term “impostor syndrome” in particular, has exploded, both in academic research articles and in popular media outlets. While this widespread attention toward impostor “syndrome” has undoubted value, including the empowerment of individuals who struggle with impostor feelings, in this perspectives article we identify critical problems with the way this phenomenon is currently being discussed and explained, both within and outside of the scientific community. Although findings are mixed regarding gender differences in impostor feelings (for a recent review see Bravata et al., 2019), both in academic literature, and in popular media outlets, the impostor phenomenon is often linked to women and members of ethnic minority groups (e.g., McGregor et al., 2008; Peteet et al., 2015). Despite impostor feelings being linked to these social groups, and the unique challenges that members of these groups face (Cokley et al., 2015, 2017; Lige et al., 2017; Bernard et al., 2018), we show that scholars have predominantly depicted and empirically examined the phenomenon at the individual level of analysis (e.g., Bernard et al., 2002; Rohrmann et al., 2016; Bravata et al., 2019). More importantly, we identify significant limitations that arise from this tendency to over-individualize the impostor phenomenon.

Next, we propose a shift in the way scholars conceptualize and empirically study, the impostor phenomenon. Instead of framing the insecurities of individuals, especially those who belong to marginalized groups, as a problem that arises from *within* these individuals, we attest that it is time that researchers consider the important role of outside forces as well—how context and social structure *create* impostor feelings. In this way, we contend that people’s impostor feelings are not solely a result of their dispositions and personalities, but can actually work their way from the outside in (see also McElwee and Yurak, 2010; Cohen and McConnell, 2019). These internalized, negative perceptions of the self are borne out of environments and social interactions that *lead people* to question their abilities and worth. Finally, based on our analysis of how social context, at multiple levels, can affect impostor feelings, we provide concrete directions for future research, and discuss implications for how to more effectively and *collectively* combat impostor feelings.

## Clinical-Psychological Perspective

With its roots in clinical psychology, scholars have predominantly depicted the impostor phenomenon as a personality trait that originates within the individuals who experience impostor feelings (for a recent review, see Bravata et al., 2019). This focus on the individual level of analysis is most likely the result of the fact that the phenomenon is reflective of a negative and critical self-concept (Clance and Imes, 1978) and negatively affects the individuals who experience it (e.g., Sonnak and Towell, 2001; McGregor et al., 2008). As such, both the experience and outcomes of the impostor phenomenon occur at the level of the individual, making this the primary focus of scholars.

This individualistic approach is, for instance, apparent in the terminology that is often used to describe the impostor phenomenon. While Clance and Imes. (1978) coined the term “impostor phenomenon,” both academic research and public discourse typically refer to it as the “impostor syndrome,” strengthening the seemingly individual, and dysfunctional, nature of the phenomenon (e.g., Kets de Vries, 1990; Rohrmann et al., 2016; Bravata et al., 2019). Depicting the phenomenon as a “syndrome,” gives the impression that the individuals who experience it are “patients” (Bravata et al., 2019, p. 1), which is highly problematic, as it implies a medical model of dysfunction within the individual (American Psychiatric Association, 2013; see also Kolligan and Sternberg, 1991).

This individual level understanding of impostor feelings is also reflected in empirical work on the phenomenon. This line of research has, for instance, primarily operationalized and measured the impostor phenomenon as a trait (instead of a state; Harvey, 1981; Clance, 1985; Mak et al., 2019). Moreover, scholars have predominantly pointed toward the individual to understand the roots and causes of impostor feelings. In this regard, researchers have, for example, regarded individuals’ attachment styles (e.g., Sonnak and Towell, 2001; Gibson-Beverly and Schwartz, 2008), perfectionistic tendencies (e.g., Henning et al., 1998; Dudãu, 2014) and personality (Harvey, 1981; Bernard et al., 2002; Vergauwe et al., 2014) as antecedents of impostor feelings.

Because of this individualistic, person-based approach to the impostor phenomenon, many of the proposed solutions and strategies for addressing impostor feelings nowadays focus on trying to “fix” the individual. Solutions in this regard typically involve clinical therapy (Langford and Clance, 1993), coaching and confidence training (Zanchetta et al., 2020) and entreaties to “lean in” (Sandberg, 2013) and “overcome” one’s own impostor “syndrome” (Dickerson, 2019). At best, the efficacy of these solutions is likely to fall short of its full potential, in part because such an approach misses an important piece of the puzzle—how the social context may shape one’s tendency to feel like an impostor. At worst, such individual-focused solutions can reinforce notions of victim blaming, whereby others perceive that the “problem” is with the individual, and so the individual is also ultimately responsible for “fixing” their own situation (Crawford, 1977; Janoff-Bulman et al., 1985; Niemi and Young, 2016).

## Social-Psychological Perspective

We argue that to fully understand the impostor phenomenon it is therefore important to complement the previous line of work at the individual level of analysis, by adding a contextual perspective on impostor feelings (see also Heller et al., 2007). Although the phenomenon manifests at the level of the individual, that is, individuals feel like impostors, these individuals do not exist in a social vacuum. Instead, people’s social context is of great importance in determining how they feel about themselves (e.g., Tajfel and Turner, 1986; Hogg and Terry, 2000). Drawing from a rich body of social psychological work, we therefore believe that there is utility in expanding the scope of impostor research to also consider the role of social context in shaping individuals’ impostor feelings.

Importantly, while Clance considered the possibility that impostor feelings might be shaped by “interpersonal and social contexts” (Clance et al., 1995, p. 80), scholars and practitioners have yet to give the social roots of this phenomenon the theoretical and empirical attention it deserves. Considering the detrimental consequences of the impostor phenomenon, it seems particularly important to systematically examine contextual factors as well—in order to gain a more complete understanding of the contextual roots that underlie this pervasive phenomenon. Building on a rich body of social-psychological work, we therefore consider how (a) society and culture at large, (b) organizations and other institutions, and (c) everyday interactions and interpersonal relationships, may play critical roles in shaping impostor feelings.

### Societal-Level Explanation

At the societal level, research suggests that an individual’s position in the social hierarchy can play an important role in shaping his or her impostor feelings, including how the specific challenges and stressors that accompany a lower societal position can make one feel like an impostor (Cokley et al., 2013; McClain et al., 2016; Chrousos et al., 2020; Chrousos and Mentis, 2020). We know from abundant social-psychological research that those groups in society that are often linked to the impostor “syndrome,” such as women and ethnic minorities, are also subject to persistent negative stereotyping (Eagly et al., 2000; Eagly and Karau, 2002; Ellemers, 2018). For example, because of the stereotype of the “good” leader possessing predominantly masculine traits (Powell et al., 2002), women are often depicted as lacking leadership qualities (i.e., they are stereotypically perceived as communal and warm), while men are portrayed as having a more natural fit for leadership positions (i.e., they are stereotypically perceived as agentic and assertive; Heilman, 2001). In response to these gender and leader stereotypes, research suggests that a woman may feel insecure and out of place if she were to achieve such a leadership position, as these pervasive stereotypes have consistently signaled, both directly and indirectly, that she would not be fit for such a position (Heilman, 2012; Haynes and Heilman, 2013). First research indeed suggests that a woman’s awareness of such stereotypes can trigger her to feel like an impostor (Cokley et al., 2015). This may also help explain inconsistencies in the current literature regarding gender differences in impostor feelings (with some studies showing that women experience more impostor feelings, while other studies failed to find gender differences; Bravata et al., 2019), as our reasoning suggests that women would only feel like impostors in contexts that signal that they are so.

Likewise, certain ethnic minorities are stereotyped as being unintelligent, lazy, and/or underachieving (Reyna, 2000, 2008). In response to such negative portrayals of their group, ethnic minority students are likely to worry that their admission to, for instance, a prestigious university is the outcome of luck, instead of something they actually deserve. In line with such reasoning, research examining impostor feelings among ethnic minority students indeed showed that students who reported being racially discriminated against were more likely to feel like impostors (Austin et al., 2009; Cokley et al., 2017; Bernard et al., 2018). Overall, this suggests that, at the societal level, the group that someone belongs to, and the portrayal of those groups in society, play an important role in triggering individuals’ impostor feelings.

### Institutional-Level Explanation

In addition to the broader societal context, research from social and organizational psychology suggests that features within the more immediate institutional context (e.g., within corporate organizations, educational or government institutions) play an important role in shaping impostor feelings as well. Here, women and ethnic minority group members are, for instance, more or less likely to occupy particular professions (e.g., they are under-represented in surgery, but over-represented in nursing), particular roles within an organization (e.g., they are under-represented in information technology, but over-represented in human resources), and particular levels of organizational hierarchies (e.g., they are under-represented in leadership positions, but over-represented at more junior levels; Catalyst, 2018). Moreover, they often lack role models and are paid less for the work they do (Lyness and Thompson, 2000; Catalyst, 2018). Research suggests that such a lack of representation and lower compensation, in turn, elicit doubts about one’s suitability for these occupations and positions (Peters et al., 2012). This line of research therefore suggests that institutional structures may cause women and ethnic minority group members to question their “place” within certain institutions (traditionally occupied by white men), thereby increasing their susceptibility to feel like “impostors” when in those institutions.

### Interpersonal-Level Explanation

Finally, social psychological research suggests that how people are treated by self-relevant others is an important precursor to impostor feelings. This is because individuals’ everyday interactions are laced with important social evaluative cues, conveying whether others see them as a person of value and worth (Smith et al., 1998; Huo and Binning, 2008). These social evaluative cues ultimately guide individuals’ appraisals of their own self-worth, and thus shape their self-esteem and sense of being worthy or deserving of their “place” within that group or context (Lind and Tyler, 1988). In traditionally white, male-dominated occupations, for example, female and ethnic minority employees are often perceived and treated differently (e.g., they are less often sought out for advice, or included in work-related discussions; Dovidio et al., 1986; Begeny et al., 2020). Such subtle everyday oversights communicate that these employees’ ideas, knowledge, and insights are valued less as that of other employees, which can in turn perpetuate issues of confidence and engagement at work (Holleran et al., 2011). Thus, to fully understand individuals’ impostor feelings, this research suggests that it is key to consider the quality of treatment people receive from others– particularly the types of treatment that communicates a sense of value, worth, and fit. In this regard, we contend that individuals may very well feel like impostors when they are treated in ways to suggest they are. Similarly, such impostor feelings can also be mitigated, when these individuals are treated by others as a person of value and worth.

## Discussion

In this article, we presented an alternative perspective on the impostor phenomenon. Although there are personal differences in the extent to which people feel like impostors, we have shown that there is considerable theoretical and conceptual support for the notion that the impostor phenomenon is also context dependent. This perspective provides an important new angle for future research: instead of focusing heavily on characteristics of the individual, we urge future research to examine contextual variables at the societal, institutional, and interpersonal levels, which may shape an individual’s impostor feelings. For example, scholars could examine whether organizations and institutions implementing diversity initiatives or affirmative action plans (Kravitz, 2008) will see a decline in impostor feelings among their members. Furthermore, future researchers could conduct interventions that aim to directly increase organizational members’ sense of fit with, or belonging to, their organization (Peters et al., 2013; Binning et al., 2020), to test whether such an intervention in turn reduces impostor feelings. Similarly, in experimental settings, researchers could manipulate interpersonal treatment (e.g., Porath and Erez, 2007) to examine its causal impact on individuals’ impostor feelings. When pursuing this important line of work, we also suggest that it will be important for researchers to be cautious with the terminology they adopt. In this regard, we feel that the original term “impostor phenomenon” is preferred over “impostor syndrome,” as the former better captures the complexity and multi-dimensional origins of the construct.

Our proposed perspective also has important implications for how to combat impostor feelings. Instead of focusing on how individuals themselves should battle their impostor feelings (Sandberg, 2013; Dickerson, 2019; Zanchetta et al., 2020), our perspective outlines the importance of addressing the contextual roots of this phenomenon—by tackling persistent stereotypes in society, increasing diversity across occupations and hierarchical levels, and assuring equal treatment for all group members. Such contextual interventions—as opposed to more individualized treatments—might also have the benefit of *preventing* impostor feelings, as opposed to merely combatting them once they emerge. Thus, if organizations can challenge societal norms and stereotypes, provide an organizational culture that reinforces feelings of inclusiveness and fit within the organization, and have clear standards of equal and inclusive treatment in the workplace, members of minority groups will be more likely to respond with lower incidences of impostor feelings.

Overall, our aim with this perspectives article is to help refocus the way in which both scholars and the broader public consider the roots and solutions of the impostor phenomenon. Our hope is that they will come to see the impostor phenomenon as not merely a dysfunctional “syndrome” that resides within certain individuals, but instead as a psychological response to a dysfunctional context. Consequently, we hope to spur future research that examines the role of individuals’ social context in shaping their impostor feelings. Overall, such a shift in thinking about, and empirically examining, the impostor phenomenon, has the potential to lead to *systemic* change, which will create an environment in which everyone feels as though they rightly belong.

## Data Availability Statement

The original contributions presented in the study are included in the article/supplementary material, further inquiries can be directed to the corresponding author.

## Author Contributions

SF: conceptualization and writing (original draft). CB and MR: conceptualization and writing (review and editing). FR, JS, and JJ: writing (review and editing). All authors contributed to the article and approved the submitted version.

## Conflict of Interest

The authors declare that the research was conducted in the absence of any commercial or financial relationships that could be construed as a potential conflict of interest.

## References

[B1] American Psychiatric Association. (2013). *Diagnostic and Statistical Manual of Mental Disorders*, 5th Edn Arlington: American Psychiatric Association.

[B2] Austin C. C. Clark E. M. Ross M. J. Taylor M. J. (2009). Impostorism as a mediator between survivor guilt and depression in a sample of African American college students. *College Stud. J.* 43 1094–1109.

[B3] Begeny C. T. Ryan M. K. Moss-Racusin C. A. Ravetz G. (2020). In some professions women have become well-represented, yet gender bias persists–perpetuated by those who think it is not happening. *Sci. Adv.* 6:eaba7814 10.1126/sciadv.aba7814 PMC7319752 32637616

[B4] Bernard D. L. Hoggard L. S. Neblett E. W. Jr. (2018). Racial discrimination, racial identity, and impostor phenomenon: a profile approach. *Cultur. Divers. Ethnic Minor. Psychol.* 24 51–61. 10.1037/cdp0000161 28414495

[B5] Bernard N. S. Dollinger S. J. Ramaniah N. V. (2002). Applying the big five personality factors to the impostor phenomenon. *J. Pers. Assess.* 78 321–333. 10.1207/s15327752jpa7802_07 12067196

[B6] Binning K. R. Kaufmann N. McGreevy E. M. Fotuhi O. Chen S. Marshman E. (2020). Changing social contexts to foster equity in college science courses: an ecological-belonging intervention. *Psychol. Sci.* 31 1059–1070. 10.1177/0956797620929984 32845825

[B7] Bravata D. M. Watts S. A. Keefer A. L. Madhusudhan D. K. Taylor K. T. Clark D. M. (2019). Prevalence, predictors, and treatment of impostor syndrome: a systematic review. *J. Gen. Intern. Med.* 35 1252–1275. 10.1007/s11606-019-05364-1 31848865 PMC7174434

[B8] Catalyst (2018). *Quick take: Women in the workforce–Global.* Available online at: https://www.catalyst.org/research/women-in-the-workforce-global/ (accessed January 30, 2020).

[B9] Chrousos G. P. Mentis A. A. (2020). Imposter syndrome threatens diversity. *Nature* 367 749–750. 10.1126/science.aba8039 32054753

[B10] Chrousos G. P. Mentis A. A. Dardiotis E. (2020). Focusing on the neuro-psycho-biological and evolutionary underpinnings of the imposter syndrome. *Front. Psychol.* 11:1553 10.3389/fpsyg.2020.01553 PMC7396514 32848987

[B11] Clance P. R. (1985). *The Impostor Phenomenon: Overcoming The Fear That Haunts Your Success*, Vol. 209 Atlanta, GA: Peachtree Publishers.

[B12] Clance P. R. Dingman D. Reviere S. L. Stober D. R. (1995). Impostor phenomenon in an interpersonal/social context: origins and treatment. *Women Ther.* 16 79–96. 10.1300/j015v16n04_07

[B13] Clance P. R. Imes S. A. (1978). The imposter phenomenon in high achieving women: dynamics and therapeutic intervention. *Psychother. Theory Res. Pract.* 15:241 10.1037/h0086006

[B14] Cohen E. D. McConnell W. R. (2019). Fear of fraudulence: graduate school program environments and the impostor phenomenon. *Sociol. Q.* 60 457–478. 10.1080/00380253.2019.1580552

[B15] Cokley K. Awad G. Smith L. Jackson S. Awosogba O. Hurst A. (2015). The roles of gender stigma consciousness, impostor phenomenon and academic self-concept in the academic outcomes of women and men. *Sex Roles* 73 414–426. 10.1007/s11199-015-0516-7

[B16] Cokley K. McClain S. Enciso A. Martinez M. (2013). An examination of the impact of minority status stress and impostor feelings on the mental health of diverse ethnic minority college students. *J. Multicult. Counse. Devel.* 41 82–95. 10.1002/j.2161-1912.2013.00029.x

[B17] Cokley K. Smith L. Bernard D. Hurst A. Jackson S. Stone S. (2017). Impostor feelings as a moderator and mediator of the relationship between perceived discrimination and mental health among racial/ethnic minority college students. *J. Couns. Psychol.* 64 141–154. 10.1037/cou0000198 28277731

[B18] Crawford R. (1977). You are dangerous to your health: the ideology and politics of victim blaming. *Int. J. Health Serv.* 7 663–680. 10.2190/YU77-T7B1-EN9X-G0PN 410739

[B19] Kets de Vries M. F. R. (1990). The impostor syndrome: developmental and societal issues. *Human Relat.* 43 667–686. 10.1177/001872679004300704

[B20] Kets de Vries M. F. R. (2005). The dangers of feeling like a fake. *Harv. Bus. Rev.* 83:108. 16171215

[B21] Dickerson D. (2019). How I overcame impostor syndrome after leaving academia. *Nature* 574 588–588. 10.1038/d41586-019-03036-y 31641254

[B22] Dovidio J. F. Evans N. Tyler R. B. (1986). Racial stereotypes: the contents of their cognitive representations. *J. Exp. Soc. Psychol.* 22 22–37., 10.1016/0022-1031(86)90039-9

[B23] Dudãu D. P. (2014). The relation between perfectionism and impostor phenomenon. *Procedia Soc. Behav. Sci.* 127 129–133. 10.1016/j.sbspro.2014.03.226

[B24] Eagly A. H. Karau S. J. (2002). Role congruity theory of prejudice toward female leaders. *Psychol. Rev.* 109 573–598. 10.1037/0033-295x.109.3.573 12088246

[B25] Eagly A. H. Wood W. Diekman A. B. (2000). “Social role theory of sex differences and similarities: a current appraisal,” in *The Developmental Social Psychology of Gender*, eds Eckes T. Trautner H. M. (Mahwah, NJ: Lawrence Erlbaum Associates Publishers), 123–174.

[B26] Ellemers N. (2018). Gender stereotypes. *Annu. Rev. Psychol.* 69 275–298. 10.1146/annurev-psych-122216-011719 28961059

[B27] Gibson-Beverly G. Schwartz J. P. (2008). Attachment, entitlement, and the impostor phenomenon in female graduate students. *J. Coll. Couns.* 11 119–132. 10.1002/j.2161-1882.2008.tb00029.x

[B28] Harvey J. C. (1981). The impostor phenomenon and achievement: a failure to internalize success. *Diss. Abstr. Int.* 42 4969–4970.

[B29] Haynes M. C. Heilman M. E. (2013). It had to be you (not me)!: women’s attributional rationalization of their contribution to successful joint work outcomes. *Pers. Soc. Psychol. Bull.* 39 956–969. 10.1177/0146167213486358 23653067

[B30] Heilman M. E. (2001). Description and prescription: how gender stereotypes prevent women’s ascent up the organizational ladder. *J. Soc. Issues* 57 657–674. 10.1111/0022-4537.00234

[B31] Heilman M. E. (2012). Gender stereotypes and workplace bias. *Res. Organ. Behav.* 32 113–135. 10.1016/j.riob.2012.11.003

[B32] Heller D. Watson D. Komar J. Min J.-A. Perunovic W. Q. E. (2007). Contextualized personality: traditional and new assessment procedures. *J. Pers.* 75 1229–1254. 10.1111/j.1467-6494.2007.00474.x 17995464

[B33] Henning K. Ey S. Shaw D. (1998). Perfectionism, the impostor phenomenon and psychological adjustment in medical, dental, nursing and pharmacy students. *Med. Educ.* 32 456–464. 10.1046/j.1365-2923.1998.00234.x 10211285

[B34] Hogg M. A. Terry D. J. (2000). Social identity and self-categorization processes in organizational contexts. *Acad. Manage. Rev.* 25 121–140. 10.2307/259266

[B35] Holleran S. E. Whitehead J. Schmader T. Mehl M. R. (2011). Talking shop and shooting the breeze: a study of workplace conversation and job disengagement among STEM faculty. *Soc. Psychol. Pers. Sci.* 2 65–71. 10.1177/1948550610379921

[B36] Huo Y. J. Binning K. R. (2008). Why the psychological experience of respect matters in group life: an integrative account. *Soc. Pers. Psychol. Compass* 2 1570–1585. 10.1111/j.1751-9004.2008.00129.x

[B37] Janoff-Bulman R. Timko C. Carli L. L. (1985). Cognitive biases in blaming the victim. *J. Exp. Soc. Psychol.* 21 161–177. 10.1016/0022-1031(85)90013-7

[B38] Kolligan J. Sternberg R. J. (1991). Perceived fraudulence in young adults: is there an “imposter syndrome”? *J. Pers. Assess.* 56 308–326. 10.1207/s15327752jpa5602_10 2056424

[B39] Kravitz D. A. (2008). The diversity-validity dilemma: beyond selection–The role of affirmative action. *Pers. Psychol.* 61 173–193. 10.1111/j.1744-6570.2008.00110.x

[B40] Langford J. Clance P. R. (1993). The imposter phenomenon: recent research findings regarding dynamics, personality and family patterns and their implications for treatment. *Psychother. Theory Res. Pract. Train.* 30 495–501. 10.1037/0033-3204.30.3.495

[B41] Lige Q. M. Peteet B. J. Brown C. M. (2017). Racial identity, self-esteem, and the impostor phenomenon among African American college students. *J. Black Psychol.* 43 345–357. 10.1177/0095798416648787

[B42] Lind E. A. Tyler T. R. (1988). *The Social Psychology Of Procedural Justice.* New York, NY: Plenum Press.

[B43] Lyness K. S. Thompson D. E. (2000). Climbing the corporate ladder: do female and male executives follow the same route? *J. Appl. Psychol.* 85 86–101. 10.1037/0021-9010.85.1.86 10740959

[B44] Mak K. K. L. Kleitman S. Abbott M. J. (2019). Impostor phenomenon measurement scales: a systematic review. *Front. Psychol.* 10:671. 10.3389/fpsyg.2019.00671 31024375 PMC6463809

[B45] McClain S. Beasley S. T. Jones B. Awosogba O. Jackson S. Cokley K. (2016). An examination of the impact of racial and ethnic identity, impostor feelings, and minority status stress on the mental health of black college students. *J. Multicult. Counse. Dev.* 44 101–117. 10.1002/jmcd.12040

[B46] McElwee R. O. Yurak T. J. (2010). The phenomenology of the impostor phenomenon. *Individ. Dif. Res.* 8 184–197.

[B47] McGregor L. N. Gee D. E. Posey K. E. (2008). I feek like a fraud and it depresses me: the relationship between the imposter phenomenon and depression. *Soc. Behav. Pers. Int. J.* 36 43–48. 10.2224/sbp.2008.36.1.43

[B48] Neureiter M. Traut-Mattausch E. (2016). Inspecting the dangers of feeling like a fake: an empirical investigation of the impostor phenomenon in the world of work. *Front. Psychol.* 7:1445. 10.3389/fpsyg.2016.01445 27729882 PMC5037221

[B49] Niemi L. Young L. (2016). When and why we see victims as responsible: the impact of ideology on attitudes toward victims. *Pers. Soc. Psychol. Bull.* 42 1227–1242. 10.1177/0146167216653933 27340155

[B50] Peteet B. J. Montgomery L. Weekes J. C. (2015). Predictors of imposter phenomenon among talented ethnic minority undergraduate students. *J. Negro Educ.* 84 175–186. 10.7709/jnegroeducation.84.2.0175 15934771

[B51] Peters K. Haslam S. A. Ryan M. K. Fonseca M. (2013). Working with subgroup identities to build organizational identification and support for organizational strategy: a test of the ASPIRe Model. *Group Organ. Manag.* 38 128–144. 10.1177/1059601112472368

[B52] Peters K. Ryan M. Haslam S. A. Fernandes H. (2012). To belong or not to belong. *J. Pers. Psychol.* 11 148–158. 10.1027/1866-5888/a000067

[B53] Porath C. L. Erez A. (2007). Does rudeness really matter? The effects of rudeness on task performance and helpfulness. *Acad. Manag. J.* 50 1181–1197. 10.5465/amj.2007.20159919

[B54] Powell G. N. Butterfield D. A. Parent J. D. (2002). Gender and managerial stereotypes: have the times changed? *J. manage.* 28 177–193. 10.1177/014920630202800203

[B55] Reyna C. (2000). Lazy, dumb, or industrious: when stereotypes convey attribution information in the classroom. *Educ. Psychol. Rev.* 12 85–110. 10.1023/A:1009037101170

[B56] Reyna C. (2008). Ian is intelligent but Leshaun is lazy: antecedents and consequences of attributional stereotypes in the classroom. *Eur. J. Psychol. Educ.* 23 439–458. 10.1007/BF03172752

[B57] Rohrmann S. Bechtoldt M. N. Leonhardt M. (2016). Validation of the impostor phenomenon among managers. *Front. Psychol.* 7:821. 10.3389/fpsyg.2016.00821 27313554 PMC4890534

[B58] Sandberg S. (2013). *Lean in: Women, Work, and the Will to Lead.* New York, NY: Alfred A. Knopf.

[B59] Smith H. J. Tyler T. R. Huo Y. J. Ortiz D. J. Lind E. A. (1998). The self-relevant implications of the group-value model: group membership, self-worth, and treatment quality. *J. Exp. Soc. Psychol.* 34 470–493. 10.1006/jesp.1998.1360

[B60] Sonnak C. Towell T. (2001). The impostor phenomenon in British university students: relationships between self-esteem, mental health, parental rearing style and socioeconomic status. *Pers. Indiv. Dif.* 31 863–874. 10.1016/S0191-8869(00)00184-7

[B61] Tajfel H. Turner J. C. (1986). “The social identity theory of inter-group behavior,” in *Psychology of intergroup relations*, eds Worchel S. Austin W. G. (Chicago, IL: Nelson-Hall), 7–24.

[B62] Vergauwe J. Wille B. Feys M. De Fruyt F. Anseel F. (2014). Fear of being exposed: the trait-relatedness of the impostor phenomenon and its relevance in the work context. *J. Bus. Psychol.* 30 565–581. 10.1007/s10869-014-9382-5

[B63] Zanchetta M. Junker S. Wolf A.-M. Traut-Mattausch E. (2020). “Overcoming the fear that haunts your success”–The effectiveness of interventions for reducing the impostor phenomenon. *Front. Psychol.* 11:405. 10.3389/fpsyg.2020.00405 32499733 PMC7242655

